# Is It Inflammatory Bowel Disease Flare or Pediatric Inflammatory Multisystem Syndrome Temporally Associated with COVID-19?

**DOI:** 10.3390/jcm11102765

**Published:** 2022-05-13

**Authors:** Paulina Krawiec, Violetta Opoka-Winiarska, Elżbieta Pac-Kożuchowska

**Affiliations:** 1 Department of Pediatrics and Gastroenterology, Medical University of Lublin, Racławickie 1, 20-059 Lublin, Poland; elzbietapackozuchowska@umlub.pl; 2 Department of Pediatric Pulmonology and Rheumatology, Medical University of Lublin, Racławickie 1, 20-059 Lublin, Poland; violettaopokawiniarska@umlub.pl

**Keywords:** SARS-CoV-2, COVID-19, PIMS-TS, MIS-C, Crohn’s disease, ulcerative colitis, children

## Abstract

Background: Pediatric inflammatory multisystem syndrome temporally associated with COVID-19/multi-system inflammatory syndrome in children (PIMS-TS/MIS-C) is a potentially life-threatening complication of SARS-CoV-2 infection in children. Gastrointestinal manifestations are prominent in children with PIMS-TS/MIS-C. Thus, it is challenging to differentiate this condition from an exacerbation of inflammatory bowel disease (IBD). We aimed to present the clinical characteristics, and diagnostic and therapeutic difficulties in patients with overlapping IBD and PIMS-TS/MIS-C; Methods: We reviewed medical records of children hospitalized due to overlapping IBD and PIMS-TS/MIS-C in a single pediatric hospital from December 2020 to December 2021; Results: There were four children with overlapping IBD flare and PIMS-TS/MIS-C. In three cases, IBD recognition preceded PIMS-TS/MIS-C onset and PIMS-TS/MIS-C occurred during anti-inflammatory therapy of IBD. All children presented with gastrointestinal symptoms at PIMS-TS/MIS-C onset. All patients received IVIG and ASA treatment. In three children there was a need to use steroids to resolve PIMS-TS/MIS-C symptoms. One child was vaccinated against COVID-19; Conclusions: SARS-CoV-2 infection may affect patients with underlying inflammatory conditions such as IBD, inducing systemic symptoms of PIMS-TS/MIS-C, and probably triggering IBD after PIMS-TS/MIS-C. The resemblance of clinical presentations is the main source of diagnostic and therapeutic challenges in PIMS-TS/MIS-C in patients with underlying IBD.

## 1. Introduction

Pediatric inflammatory multisystem syndrome temporally associated with COVID-19/multi-system inflammatory syndrome in children (PIMS-TS/MIS-C) is a rare and potentially life-threatening complication of SARS-CoV-2 infection in children [1]. Different case definitions of PIMS-TS/MIS-C have been released by the Royal College of Pediatrics and Child Health, World Health Organization and Centers for Disease Control and Pre-vention [2,3,4].

The clinical spectrum of PIMS-TS/MIS-C is heterogeneous. It may include various and non-specific symptoms from almost any organ and system [5]. However, it has been shown that gastrointestinal manifestations are particularly frequent and prominent in children with PIMS-TS/MIS-C [6]. Thus, it may be challenging to differentiate this condition from an exacerbation of chronic inflammatory conditions including inflammatory bowel disease (IBD) [7,8]. Herein we aimed to present clinical characteristics and difficulties in the management of children with concurrent IBD and PIMS-TS/MIS-C based on our experience with four patients.

## 2. Materials and Methods

We reviewed retrospectively the medical records of children hospitalized due to overlapping IBD and PIMS-TS/MIS-C in the Children’s University Hospital in Lublin, Poland from December 2020 to December 2021. The diagnosis of IBD was made based on the European Society of Pediatric Gastroenterology, Hepatology and Nutrition (ESPGHAN) revised Porto criteria [9]. The phenotype of IBD was determined using the Paris classification [9,10].

Clinical activity of IBD was established based on Pediatric Crohn’s Disease Activity Index (PCDAI) or Pediatric Ulcerative Colitis Activity Index (PUCAI). The higher scores, the more severe the clinical activity of IBD. The sum of PCDAI scores ranges from 0 to 100. According to the PCDAI, clinical remission of Crohn’s disease is defined as ≤10 points while the active phase is >10 points. PUCAI scores range from 0 to 85. According to the PUCAI, the score of remission of ulcerative colitis is defined as less than 10 points and the active phase as ≥10 points [11,12].

PIMS-TS/MIS-C was recognized based on the case definition by the Royal College of Pediatrics and Child Health, the World Health Organization, and the Centers for Disease Control and Prevention [2,3,4].

We collected data on patients’ age, sex, signs and symptoms, results of laboratory, and imaging tests at PIMS-TS/MIS-C onset. We analyzed the course of PIMS-TS/MIS-C and follow-up health outcomes including the course of IBD. The study was approved by the Bioethics Committee at the Medical University of Lublin (No. KE-0254-116-2020).

## 3. Results

From December 2020 to December 2021 there were four children hospitalized due to overlapping IBD and PIMS-TS/MIS-C in the Children’s University Hospital in Lublin, Poland. At that time, PIMS-TS/MIS-C was diagnosed in our hospital in over 80 children. In about 80% of them, the first symptoms were abdominal pain, vomiting, and diarrhea which occurred prior to or concurrently with fever.

We present a brief description of the natural history of IBD and PIMS-TS/MIS-C in these patients below. Table 1 summarizes the basic clinical characteristics of our patients. Table 2 presents selected laboratory results of our patients at the time of PIMS-TS/MIS-C recognition.

### 3.1. Case 1

A 14-year-old boy with Crohn’s disease, diagnosed in May 2020, was admitted to our hospital with a one-week history of fever and diarrhea.

Medical history: Initially the patient was diagnosed with IBD type unclassified. In induction therapy, oral corticosteroids were used. Then, mesalamine as maintenance treatment was used with improvement. Clinical remission was achieved for 6 months. The second flare of IBD manifested with diarrhea, abdominal pain, and weight loss occurred in December 2020. At that time repeated endoscopy and histological evaluation allowed recognition of Crohn’s disease colitis. In the treatment, exclusive enteral nutrition followed by azathioprine was started. About two weeks later the patient was admitted to our department due to a one-week history of fever up to 39 °C, severe diarrhea, and loss of weight of about 4 kg within a week.

On admission, the patient was in a fair general condition, apathetic. The temperature of the body was 38.2 °C, blood pressure 97/63 mmHg. Abnormalities in physical examination included bilateral non-purulent conjunctivitis, strawberry tongue (prominent lingual papillae), erythematous and cracked lips, rash on extremities and trunk, erythema nodosum on both lower extremities, tachycardia 110 beats per minute.

Laboratory results revealed microcytic anemia, lymphopenia, thrombocytosis, elevated CRP and ESR, hyponatremia, high fibrinogen level, elevated D-dimers, increase in amylase and lipase. Nasopharyngeal swab for SARS-CoV-2 by RT-PCR was negative. Antibodies against SARS-CoV-2 were positive in IgG and negative in IgM.

Ultrasound examination of the abdomen showed a slightly enlarged liver and increased thickness of the sigmoid and descending colon wall. Chest X-ray was normal. The cardiovascular evaluation did not reveal cardiac involvement.

The patient was recognized with a flare of IBD complicated by PIMS-TS/MIS-C. In accordance with the recommendations [13,14], he received intravenous immunoglobulin infusion (IVIG), acetylsalicylic acid (ASA), and an antibiotic (ceftriaxone). The treatment of Crohn’s disease was modified, i.e., enteral nutrition was ceased, steroid therapy was initiated, and azathioprine was continued. We observed an improvement in the patient’s signs and symptoms. There were no cardiological complications of PIMS-TS/MIS-C. Within a one-year follow-up period, the patient experienced another exacerbation of Crohn’s disease and he was switched from azathioprine to methotrexate with improvement. Currently, he is in clinical remission on methotrexate therapy.

### 3.2. Case 2

A 10-year-old girl was diagnosed with ulcerative colitis in November 2020. She received oral corticosteroids and mesalamine to induce remission, which resulted in an improvement in clinical symptoms. About a month after IBD onset, during steroids tapering in December 2020, the girl was admitted to the hospital due to fever, non-bloody diarrhea, myalgia, chest pain, and progressive fatigue which started a day before admission.

On admission the patient was in a fair general condition, complaining of chest pain. Her vital signs were as follows: HR 150 bpm, BP 101/68 mmHg, temperature 38.5 °C. In physical examination, we found tachycardia and tenderness of the abdomen.

Laboratory results revealed elevated inflammatory markers, minor anemia, elevated troponin, ferritin, CK-MB, NT-pro-BNP, and D-dimers. Antibodies against SARS-CoV-2 were positive in IgG and negative in IgM. Electrocardiogram revealed nonspecific ST-T waves changes and sinus tachycardia. Echocardiogram showed reduced left ventricular function (left ventricular ejection fraction 53.7% and shortening fraction 27.4%). Ultrasound examination of the abdomen showed increased thickness of the ascending colon wall, minor splenomegaly, and minor dilatation of intrahepatic bile ducts.

PIMS-TS/MIS-C was recognized with myocarditis as a prominent manifestation. The patient received IVIG and ASA as recommended [13,14], which resulted in symptom resolution. However, within a fourteen-month follow-up period, the patient experienced a relapse of myocarditis, an episode of acromioclavicular arthritis, and a second flare of IBD. She is currently in clinical remission of IBD on azathioprine therapy. About a year after PIMS-TS/MIS-C, the patient was vaccinated against COVID-19 with no significant adverse reactions.

### 3.3. Case 3

A 16-year-old boy was admitted to our hospital in June 2021 due to bloody diarrhea, fever, and cough for about a week. The boy was also complaining of difficulties with swallowing and rhinitis. His past medical history was unremarkable. Abnormalities on physical examination included eyelid oedema, bilateral non-purulent conjunctivitis, strawberry tongue, swollen lips, and oral ulcerations. Laboratory results showed leukocytosis with lymphopenia, significantly elevated ESR and C-reactive protein, elevated ferritin, D-dimers, mild elevation of alanine aminotransferase, and γ-glutamyltransferase. Antibodies against SARS-CoV-2 were positive in IgG and negative in IgM.

As PIMS-TS/MIS-C was recognized the patient received IVIG and ASA. However, there was no improvement. Thus, steroids were started resulting in the gradual resolution of signs and symptoms. A month after PIMS-TS/MIS-C onset, while steroids had been tapered, bloody diarrhea reoccurred. The patient also experienced eyelid oedema and bilateral non-purulent conjunctivitis. Based on gastroscopy, colonoscopy, and histological assessment, IBD type unclassified was recognized. Apart from steroids continuation, the patient received azathioprine and mesalamine with improvement. Within a six-month follow-up period, the patient has remained in clinical remission on azathioprine treatment.

### 3.4. Case 4

An 11-year-old boy with Crohn’s disease diagnosed in November 2020 was admitted to the hospital in December 2020 due to severe vomiting and bloody diarrhea from the day before admission. The patient had been on exclusive enteral nutrition for two weeks. On admission, the boy was apathetic with severe abdominal pain. In physical examination, we stated a tender and distended abdomen, particularly in the epigastric area and right lower quadrant. Laboratory results revealed elevated inflammatory markers, hypoalbuminemia, hyponatremia, and increased activity of γ-glutamyltransferase. Abdominal ultrasound showed edematous swelling of the ileocecal valve, and distal ileum wall thickening with low echogenicity.

Exclusive enteral nutrition was withdrawn because of its intolerance, and steroids with azathioprine were started. Due to suspicion of systemic infection overlapping on IBD flare wide-spectrum antibiotics were used. There was an initial improvement. However, while steroids have been tapered, symptoms from the gastrointestinal tract have been progressing, and fever with shivers and non-productive cough occurred. The patient presented with bilateral non-purulent conjunctivitis, erythematous and cracked lips, and pleural effusion. In laboratory tests, inflammatory markers increased. Antibodies against SARS-CoV-2 were positive in IgG and negative in IgM. Moreover, due to signs of acute pancreatitis, azathioprine had to be ceased. The patient was eventually recognized with a flare of IBD complicated by PIMS-TS/MIS-C. He received IVIG and ASA, but no significant improvement was seen. Finally, the dose of methylprednisolone was increased which resulted in symptom resolution. Within a one-year follow-up, the patient was in clinical remission of IBD on methotrexate treatment, however, he was also recognized with the overlap syndrome of autoimmune hepatitis and primary sclerosing cholangitis.

## 4. Discussion

The recent systematic reviews revealed that the majority of children with PIMS-TS/MIS-C present with gastrointestinal signs and symptoms [6,15]. Insidious and severe gastrointestinal manifestations of PIMS-TS/MIS-C may mimic abdominal surgical emergencies including acute appendicitis, gastrointestinal infections, or inflammatory bowel disease [8,16]. In some patients with PIMS-TS/MIS-C, abdominal imaging studies revealed thickening of the bowel wall particularly in the distal ileum and mesenteric fat stranding, which also resembled IBD [17,18]. Moreover, although systemic inflammation and most manifestations resolved in the majority of children with PIMS-TS/MIS-C at the six-month follow-up, persistent gastrointestinal symptoms were reported in 13% of patients [19].

On the other hand, during the COVID-19 pandemic in children with inflammatory bowel disease and acute symptoms suggesting clinical relapse of the disease, a high index of suspicion of PIMS-TS/MIS-C should be maintained. Therefore, children with severe gastrointestinal presentation require meticulous evaluation concerning the possibility of PIMS-TS/MIS-C. The reason for the significant involvement of the gastrointestinal tract in PIMS-TS/MIS-C remains unclear [20]. The gastrointestinal tract is a gateway for SARS-CoV-2, as there is a remarkable co-expression of the angiotensin-converting enzyme 2 (ACE2) and the transmembrane serine protease 2 (TMPRSS2) on the surface of the esophageal upper epithelial, gland cells, and absorptive enterocytes from the ileum and colon [21]. However, it has not been established whether gastrointestinal symptoms of PIMS-TS/MIS-C result from direct tissue damage or hyperinflammatory response [17,20].

PIMS-TS/MIS-C occurs more frequently in children without any preceding comorbidity or underlying condition [6,15]. However, it should be highlighted that overweight was reported in a quarter of children with recognition of PIMS-TS/MIS-C [6]. Although our knowledge about PIMS-TS/MIS-C is expanding, little is known about the risk factors, clinical course, treatment strategies, and outcomes of PIMS-TS/MIS-C in children with underlying chronic inflammatory conditions. Until now, there have been several case reports regarding the occurrence of PIMS-TS/MIS-C in children with underlying IBD [22,23,24,25].

Sweeny et al. [24] described a case of a 16-year-old boy with a new-onset Crohn’s disease and overlapping features of PIMS-TS/MIS-C. His initial symptoms included progressive bloody diarrhea and abdominal pain which were preceded by mild rhinorrhea two weeks earlier [24]. On admission, the patient was febrile and tachycardic. His laboratory results revealed elevation of inflammatory markers, anemia, hyperferritinemia, and elevation of D-dimers [24]. In gastroscopy, features of severe gastritis and duodenitis were seen. Interestingly, in duodenal biopsies, there were features of submucosal vasculitis [24]. Colonoscopy showed patchy moderate to severe colitis, confirmed in histopathological evaluation [24]. Moreover, anti-SARS-CoV-2 IgG antibodies were detected. The patient was treated with intravenous methylprednisolone and subsequently exclusive enteral nutrition. However, because of ongoing symptoms unresponsive to that therapy, he received intravenous immune globulin. Although there was an improvement in inflammatory markers, bloody diarrhea persisted [24]. Eventually, the patient received infliximab which resulted in the rapid resolution of his gastrointestinal symptoms [24]. The presentation of our patients also illustrates, that overlapping symptoms of IBD and PIMS-TS/MIS-C posed a significant diagnostic dilemma in clinical practice and were the source of therapeutic difficulties. We may presume that immunosuppressive therapy in our patients (case reports number 1, 2, and 4) could modify the course of PIMS-TS/MIS-C which is another reason for diagnostic challenges.

Meredith et al. [25] presented a case report of a 10-year-old girl with ulcerative colitis receiving maintenance infliximab therapy who was hospitalized due to PIMS-TS/MIS-C. However, in the child, there was a lack of PCR or serological evidence of SARS-CoV-2 infection. One possible reason for negative serological testing may be suspected interference from human anti-mouse antibodies, which may develop in patients treated with mouse monoclonal antibodies, with immunoassays [25,26].

Limited information is available about the treatment of PIMS-TS/MIS-C in children with chronic inflammatory conditions including IBD. Dolinger et al. [22] described the first case of an adolescent patient with Crohn’s disease and PIMS-TS/MIS-C who required treatment with infliximab for both entities. The authors noticed that although several therapeutic options were considered including remdesivir, tocilizumab, or immune globulin, infliximab has been chosen because of the potential positive outcome of a TNF-α blockade in both Crohn’s disease and cytokine storm associated with PIMS-TS/MIS-C [22]. Clinical improvement and decrease in Il-6, IL-8, and normalization of TNF-α in the patient suggested that infliximab may be effective in halting a hyperinflammatory response in PIMS/TS/MIS-C [22].

Alkan et al. [23] presented a case report of a 15-year-old girl with ulcerative colitis treated with infliximab who revealed PIMS-TS/MIS-C refractory to intravenous immune globulin. The patient received an infliximab infusion resulting in rapid resolution of symptoms and improvement in laboratory markers of inflammation [23].

Results of the present study and previous case reports demonstrate that recognition of PIMS-TS/MIS-C in children with IBD requires clinical acumen and detailed differential diagnostics [22,23,24,25]. The constellation of symptoms from various systems with signs of hyperinflammation in our patients with IBD (Cases 1, 2, 4) enforced recognition of PIMS-TS/MIS-C rather than solely a flare of IBD.

We speculate that in our patient, presented as Case 3, PIMS-TS/MIS-C might play a role as a trigger factor of IBD onset. Penner et al. [19] also reported one patient with persistent gastrointestinal symptoms after PIMS-TS/MIS-C in whom colonoscopy revealed patchy chronic inflammatory changes with increased lamina propria eosinophil density throughout the colon and ileum. However, there are no published data regarding the clinical course of this condition [19]. Until now, it appears that the role of PIMS-TS/MIS-C as a trigger of the IBD onset is another puzzling issue that has remained undetermined yet. It has been hypothesized that autoantibodies present in patients with PIMS-TS/MIS-C may provoke the formation of an immune complex and stimulate a cell-mediated immune response against self-tissues [27]. It may be a possible underlying mechanism of potential susceptibility to autoimmune diseases [27]. However, further studies are needed to establish the link between PIMS-TS/MIS-C and the risk for autoimmune conditions.

## 5. Conclusions

To sum up, the resemblance of clinical presentations is the main source of diagnostic and therapeutic challenges in PIMS-TS/MIS-C in patients with underlying IBD. Long-term follow-up is needed to establish the risk of autoimmune conditions in children who had recovered from PIMS-TS/MIS-C.

## Figures and Tables

**Table 1:** Basic characteristics of patients with co-occurrence of IBD and PIMS-TS/MIS-C.

Parameter	Patient 1	Patient 2	Patient 3	Patient 4
Gender	Boy	Girl	Boy	Boy
Initial diagnosis	Inflammatory bowel disease (IBD)	IBD	Pediatric inflammatory multisystem syndrome temporally associated with COVID-19/multi-system inflammatory syndrome in children (PIMS-TS/MISC)	IBD
Time from initial to the second diagnosis	8 months	1 month	1 month	3 months
Patient’s age at IBD onset (in years and months)	13 years 11 months of age	10 years of age	17 years of age	11 years 3 months of age
Patient’s age at PIMS-TS/MIS-C onset (in years and months)	14 years 7 months of age	10 years 1 months of age	16 years 11 months of age	11 years 5 months of age
IBD type	Crohn’s disease	Ulcerative colitis	IBD-unclassified	Crohn’s disease
IBD location	Colon	Pancolitis	Colon	Distal ileum, Colon, Upper GIT
Crohn’s disease behavior	Inflammatory	N/A	N/A	Inflammatory
IBD clinical activity on PIMS-TS/MIS-C onset	Pediatric Crohn’s disease activity index (PCDAI) 50 pts.	Pediatric ulcerative colitis activity index (PUCAI) 30 pts.	N/A	PCDAI 45pts.

**Table 2:** Selected laboratory results at PIMS-TS/MIS-C onset.

Parameter	Patient No 1	Patient No 2	Patient No 3	Patient No 4
SARS-CoV-2 RT-PCR test	Negative	Negative	Negative	Negative
SARS-CoV-2 serology test	IgM negative IgG positive (75.8 AU/mL)	IgM negative IgG positive (>400 AU/mL)	IgM negative IgG positive (563.77 AU/mL)	IgM negative IgG positive (212 AU/mL)
Erythrocyte sedimentation rate (ESR) (mm/h)	120	46	120	120
C-reactive protein (mg/dL)	15.19	26.45	40.18	15.91
Leukocytes (/μL)	9280	20,830	21,490	19,480
Neutrophils (%)	7.5	76.9	91.5	36.6
Lymphocytes (%)	13.5	18.8	3.6	55.6
Hb (g/dL)	10.5	12.3	12.5	9.9
Thrombocytes (/μL)	459,000	309,000	542,000	633,000
Ferritin (ng/mL)	251.6	70.4	677	542
D-dimers (ng/mL)	1528	669	1765	1416
Lactic acid dehydrogenase LDH (U/L)	420	334	383	543
Na^+^ (mmol/L)	126	134	134	126
Troponin (ng/mL)	<0.1	0.35	<0.1	<0.1
NT-pro-BNP (pg/mL)	28.75	2099	58.92	197
Fecal calprotectin (ng/g)	7920	1800	1600	378

## Data Availability

Data supporting the findings of this study are available within the article.

## References

[1] Dionne A., Son M.B.F., Randolph A.G. (2022). An Update on Multisystem Inflammatory Syndrome in Children Related to SARS-CoV-2. Pediatr. Infect. Dis. J..

[2] Royal College of Paediatrics and Child Health Guidance: Paediatric Multisystem Inflammatory Syndrome Temporally Associated with COVID-19. https://www.rcpch.ac.uk/sites/default/files/2020-05/COVID-19-Paediatric-multisystem-%20inflammatory%20syndrome-20200501.pdf.

[3] US Centers for Disease Control and Prevention Multisystem Inflammatory Syndrome in Children (MIS-C) Associated with Coronavirus Disease 2019 (COVID-19). https://emergency.cdc.gov/han/2020/han00432.asp.

[4] World Health Organization Multisystem Inflammatory Syndrome in Children and Adolescents with COVID-19: Scientific brief. https://apps.who.int/iris/handle/10665/332095.

[5] Opoka-Winiarska V., Grywalska E., Roliński J. (2021). PIMS-TS, the New Paediatric Systemic Inflammatory Disease Related to Previous Exposure to SARS-CoV-2 Infection-“Rheumatic Fever” of the 21st Century?. Int. J. Mol. Sci..

[6] Hoste L., Van Paemel R., Haerynck F. (2021). Multisystem inflammatory syndrome in children related to COVID-19: A systematic review. Eur. J. Pediatr..

[7] Assa A., Benninga M.A., Borrelli O., Broekaert I., de Carpi J.M., Saccomani M.D., Dolinsek J., Mas E., Miele E., Thomson M. (2021). Gastrointestinal Perspective of Coronavirus Disease 2019 in Children-An Updated Review. J. Pediatr. Gastroenterol. Nutr..

[8] Miller J., Cantor A., Zachariah P., Ahn D., Martinez M., Margolis K.G. (2020). Gastrointestinal Symptoms as a Major Presentation Component of a Novel Multisystem Inflammatory Syndrome in Children That Is Related to Coronavirus Disease 2019: A Single Center Experience of 44 Cases. Gastroenterology.

[9] Levine A., Koletzko S., Turner D., Escher J.C., Cucchiara S., de Ridder L., Kolho K.L., Veres G., Russell R.K., Paerregaard A. (2014). ESPGHAN revised porto criteria for the diagnosis of inflammatory bowel disease in children and adolescents. J. Pediatr. Gastroenterol. Nutr..

[10] Levine A., Griffiths A., Markowitz J., Wilson D.C., Turner D., Russell R.K., Fell J., Ruemmele F.M., Walters T., Sherlock M. (2011). Pediatric modification of the Montreal classification for inflammatory bowel disease: The Paris classification. Inflamm. Bowel Dis..

[11] Hyams J.S., Ferry G.D., Mandel F.S., Gryboski J.D., Kibort P.M., Kirschner B.S., Griffiths A.M., Katz A.J., Grand R.J., Boyle J.T. (1991). Development and validation of a pediatric Crohn’s disease activity index. J. Pediatr. Gastroenterol. Nutr..

[12] Turner D., Otley A.R., Mack D., Hyams J., De Bruijne J., Uusoue K., Walters T.D., Zachos M., Mamula P., Beaton D.E. (2007). Development, validation, and evaluation of a pediatric ulcerative colitis activity index: A prospective multicenter study. Gastroenterology.

[13] Harwood R., Allin B., Jones C.E., Whittaker E., Ramnarayan P., Ramanan A.V., Kaleem M., Tulloh R., Peters M.J., Almond S. (2021). A national consensus management pathway for paediatric inflammatory multisystem syndrome temporally associated with COVID-19 (PIMS-TS): Results of a national Delphi process. Lancet Child Adolesc. Health.

[14] Henderson L.A., Canna S.W., Friedman K.G., Gorelik M., Lapidus S.K., Bassiri H., Behrens E.M., Ferris A., Kernan K.F., Schulert G.S. (2020). American College of Rheumatology Clinical Guidance for Multisystem Inflammatory Syndrome in Children Associated with SARS-CoV-2 and Hyperinflammation in Pediatric COVID-19: Version 1. Arthritis Rheumatol..

[15] Santos M.O., Gonçalves L.C., Silva P.A.N., Moreira A.L.E., Ito C.R.M., Peixoto F.A.O., Wastowski I.J., Carneiro L.C., Avelino M.A.G. (2021). Multisystem inflammatory syndrome (MIS-C): A systematic review and meta-analysis of clinical characteristics, treatment, and outcomes. J. Pediatr..

[16] Rouva G., Vergadi E., Galanakis E. (2022). Acute abdomen in multisystem inflammatory syndrome in children: A systematic review. Acta Paediatr..

[17] Sahn B., Eze O.P., Edelman M.C., Chougar C.E., Thomas R.M., Schleien C.L., Weinstein T. (2021). Features of Intestinal Disease Associated with COVID-Related Multisystem Inflammatory Syndrome in Children. J. Pediatr. Gastroenterol. Nutr..

[18] Meshaka R., Whittam F.C., Guessoum M., Eleti S., Shelmerdine S.C., Arthurs O.J., McHugh K., Hiorns M.P., Humphries P.D., Calder A.D. (2022). Abdominal US in Pediatric Inflammatory Multisystem Syndrome Associated with COVID-19. Radiology.

[19] Penner J., Abdel-Mannan O., Grant K., Maillard S., Kucera F., Hassell J., Eyre M., Berger Z., Hacohen Y., Moshal K. (2021). 6-month multidisciplinary follow-up and outcomes of patients with paediatric inflammatory multisystem syndrome (PIMS-TS) at a UK tertiary paediatric hospital: A retrospective cohort study. Lancet Child Adolesc. Health.

[20] Chen T.H., Kao W.T., Tseng Y.H. (2021). Gastrointestinal Involvements in Children with COVID-related Multisystem Inflammatory Syndrome. Gastroenterology.

[21] Zhang H., Kang Z., Gong H., Xu D., Wang J., Li Z., Li Z., Cui X., Xiao J., Zhan J. (2020). Digestive system is a potential route of COVID-19: An analysis of single-cell coexpression pattern of key proteins in viral entry process. Gut.

[22] Dolinger M.T., Person H., Smith R., Jarchin L., Pittman N., Dubinsky M.C., Lai J. (2020). Pediatric Crohn Disease and Multisystem Inflammatory Syndrome in Children (MIS-C) and COVID-19 Treated with Infliximab. J. Pediatr. Gastroenterol. Nutr..

[23] Alkan G., Emiroglu M., Oz S.K.T., Ergani A.C., Emiroglu H.H. (2021). Infliximab: A treatment option for multisystem inflammatory syndrome in children with ulcerative colitis?. Turk. Arch. Pediatr..

[24] Sweeny K.F., Zhang Y.J., Crume B., Martz C.A., Blessing M.M., Kahn S.A. (2021). Inflammatory Bowel Disease Presenting with Concurrent COVID-19 Multisystem Inflammatory Syndrome. Pediatrics.

[25] Meredith J., Khedim C.A., Henderson P., Wilson D.C., Russell R.K. (2021). Paediatric Inflammatory Multisystem Syndrome Temporally Associated with SARS-CoV-2 [PIMS-TS] in a Patient Receiving Infliximab Therapy for Inflammatory Bowel Disease. J. Crohns Colitis.

[26] Bazydlo L.A.L., Harris N.S., Winter W.E., Dasgupta A., Sepulveda J.L. (2013). Chapter 11—Challenges in Endocrinology Testing. Accurate Results in the Clinical Laboratory.

[27] Gruber C.N., Patel R.S., Trachtman R., Lepow L., Amanat F., Krammer F., Wilson K.M., Onel K., Geanon D., Tuballes K. (2020). Mapping Systemic Inflammation and Antibody Responses in Multisystem Inflammatory Syndrome in Children (MIS-C). Cell.

